# Rice‐derived peptide AAGALPS inhibits TNF‐α‐induced inflammation and oxidative stress in vascular endothelial cells

**DOI:** 10.1002/fsn3.1354

**Published:** 2019-12-19

**Authors:** Li‐Tao Tong, Zhiyuan Ju, Liya Liu, Lili Wang, Xianrong Zhou, Tianzhen Xiao, Sumei Zhou

**Affiliations:** ^1^ Institute of Agro‐Products Processing Science and Technology Chinese Academy of Agricultural Sciences/Key Laboratory of Agro‐Products Processing Ministry of Agriculture Beijing China

**Keywords:** HUVEC, inflammation, oxidative stress, rice peptide, TNF‐α

## Abstract

Injury of vascular endothelial cell is one of the main factors triggering atherosclerosis. Peptide AAGALPS was derived from digestion and absorption product of rice α‐globulin, which was proved to prevent atherosclerosis in previous study. This study aims to investigate the potential effects of AAGALPS on improving tumor necrosis factor‐α (TNF‐α)‐stimulated human umbilical vein endothelial cells’ (HUVECs) injury. As a result, the viability of HUVECs stimulated by tumor necrosis factor‐α was significantly increased by AAGALPS in a dose‐dependent manner until 25 μg/ml. The peptide obviously reduced the levels of intercellular adhesion molecule‐1, vascular cell adhesion molecule‐1, nitric oxide, inducible nitric oxide synthase, reactive oxygen species, and malondialdehyde and increased the concentrations of glutathione peroxidase. Furthermore, AAGALPS inhibited the nuclear factor κB (NF‐κB) activation and nuclear translocation through regulating inhibitor of nuclear factor κB kinase α and inhibitor of NF‐κB. These results indicated that AAGALPS protected vascular endothelial cells through mediating inflammation and oxidative stress.

## Graphical abstract

AAGALPS as one of the bioactive peptides attenuated the TNF‐α‐induced injury; AAGALPS protected the vascular endothelial cells through mediating inflammation and oxidative stress.

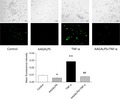

## 1 INTRODUCTION

Atherosclerotic cardiovascular diseases are the leading cause of death and disability worldwide (Bonow, 2002). Acquiring bioactive substances which have effects on resistance to atherosclerosis (AS) from natural products is becoming a research focus. Numerous studies showed that food proteins intake produced peptides with the function of preventing cardiovascular disease in intestinal. The previous study had confirmed that the AS lesion area was reduced by feeding rice α‐globulin (100 mg/kg) in the ApoE^−/−^ mice (Tong et al., 2012). However, the sequence of functional peptide and the antiatherosclerosis mechanism of rice α‐globulin are not fully understood.

Food proteins are digested into peptides, some of which are easier to be absorbed by human directly (Ingersoll et al., 2012). These peptides, used by the body, have effects on preventing lifestyle diseases, such as antiatherosclerosis, antihypertensive, antihyperlipidemia, and antioxidant (Sarmadi & Ismail, 2010). At present, sequencing of hydrolysates and synthesis from functional protein are important methods to obtain the functional peptides. The researchers have isolated antiatherogenic peptides from protein hydrolysates, such as soybean globulin peptide WH (Matsui et al., 2010), HGI, and HGK (Kumrungsee et al., 2014). Generally, absorbed protein hydrolysates were collected by everted intestinal sacs in vitro firstly. Second, the peptide components were purified by GFC and HPLC (Kim, Kawamura, & Lee, 2003). And then, the amino acid sequence of the peptides was determined by LC‐MS/MS. Finally, the peptides were compounded by solid‐phase synthesis.

Inflammation and oxidative stress are two major causes of aggravating endothelial dysfunction in atherosclerosis (Baker, Hayden, & Ghosh, 2011; Landmesser & Harrison, 2001). The nitric oxide (NO), monocyte chemotactic protein‐1 (MCP‐1), intercellular adhesion molecule‐1 (ICAM‐1), and vascular cell adhesion molecule‐1 (VCAM‐1) are important in the progress of inflammation of endothelial cells (Ahn, Xu, & Davidge, 2008; Ruimi et al., 2010; Yang et al., 2005). Moreover, ROS generated during atherosclerosis is often associated with endothelial dysfunction. Antioxidative enzymes such as superoxide dismutase (SOD) and glutathione peroxidase (GPx) are involved in the detoxification of ROS that contributes to restore oxidation–reduction equilibrium. Moreover, the pro‐inflammatory nuclear factor κB (NF‐κB) is acknowledged to one pivotal transcription factor that slows down the key steps in development of atherosclerosis (Chai, Wang, Huang, Xi, & Fu, 2008). NF‐κB is restricted to the cytosol as an inactive complex with its inhibitors such as IκBα which is phosphorylated by IκB protein kinase (IKK) and rapidly degraded. The regulation of NF‐κB may also provide a new strategy against inflammatory and oxidative stress responses. In the present study, we examined whether the peptide AAGALPS protects human umbilical vein endothelial cells (HUVECs) from the injurious effects of the pro‐inflammatory cytokine tumor necrosis factor‐alpha (TNF‐α).

## 2 MATERIALS AND METHODS

### 2.1 Materials

TNF‐α, fetal calf serum (FBS), pancreatic enzymes, and antibiotics penicillin–streptomycin were purchased from Gibco/Invitrogen. AAGALPS was synthesized by China Peptides Co. Ltd., and its purity (> 95%) was verified by HPLC‐MS/MS. Cell Counting Kit‐8 (CCK‐8) was from Dojindo Co. Ltd. Bioengineering Institute. Assay kits of reactive oxygen species (ROS), nitric oxide (NO), inducible nitric oxide synthase (iNOS), superoxide dismutase (SOD), glutathione peroxidase (GPx), and malondialdehyde (MDA) were from Expandbiotech Co., Ltd. BCA protein quantitative kit was bought from Sainobo Co., Ltd.

### 2.2 Cell culture

Human Umbilical Vein Endothelial Cells (Lot: 63505670), Vascular Cell Basal Medium, and Endothelial Cell Growth Kit‐BBE were all acquired from the ATCC. The medium was prepared according to the introduction of ATCC, containing 10% FBS, Kit‐BBE, and penicillin–streptomycin (1:1,000). HUVECs were cultured in a humidified atmosphere containing 5% CO_2_ at 37°C. After approximately 80% confluence, the cells were treated with 0.25% trypsin/1 mM EDTA. The medium containing 5% FBS was used for the cell subculture. The cells were serum‐starved overnight prior to stimulation.

### 2.3 Analysis of cell viability

Cell viability was analyzed by using CCK‐8 according to the instructions. For the CCK‐8 assay, cells were seeded in 96‐well plates (100 μL medium per well). After the density rising to 1 × 10^4^/well, cells were pretreated with the peptide (12.5, 25, 50, 100 μg/ml) for 2 hr and then stimulated with or without TNF‐α (10 ng/ml) for 6 hr in the presence of the peptide. After that, CCK‐8 solution (10 μl/well) was added and incubated for 4 hr. Cell viability was assessed by measuring the absorbance at 450 nm using a microplate reader. The absorbance is proportional to the number of viable cells in the cell culture.

### 2.4 Western blot analysis

Cells were lysed by using RIPA buffer containing trypsin inhibitor (phosphorylated proteins need to add the phosphatase inhibitor), PMSF (1 mM), and lysate (1 × 10^7^ cells/ml) on ice. Supernatant was centrifuged at 8,000 *g* for 20 min and quantified by BCA protein assay. Samples of cells lysate were resolved on SDS‐PAGE. After being transferred to membranes, the samples were immunoblotted with primary antibodies followed by secondary antibodies (goat anti‐rabbit IgG HRP (H + L)) conjugated to horseradish peroxidase. Bands were revealed using ECL Plus Western Blotting Detection Reagents (GE Healthcare) and quantified using ImageJ software. The following antibodies were used ICAM‐1 antibody (#4915), VCAM‐1 (E1E8X) rabbit mAb (#13662), MCP‐1 antibody (#2027), IKKα antibody (#2682), and IκB‐α antibody (#4812).

### 2.5 Measurement of ROS production

ROS production in cells was evaluated by fluorescent microscopic assay using 2, 7‐dichlorofluorescein diacetate (DCFH‐DA; Sigma) as a probe for the presence of superoxide. After preincubation with peptide at 25 μg/ml for 18 hr, cells in 24‐well plates were incubated with DCFH‐DA (10 μM) for 30 min, which was predissolved in DMSO, followed by the stimulation with TNF‐α (10 ng/ml) for 6 hr. Cells were washed three times using serum‐free medium to fully remove extracellular probe. The labeled cells were examined with an Olympus microscope equipped with fluorescent launcher. Photomicrographs were acquired randomly of 5 fields (×200) with an Olympus digital camera.

### 2.6 Measurement of NO content

NO is rapidly converted into the stable products nitrite and nitrate. Measurement of nitrite and nitrate was used as an indirect measurement of the amount of NO produced by HUVECs. Cells in 6‐well plates were pretreated with or without the peptide (25 μg/ml) for 2 hr before the stimulation with TNF‐α (10 ng/ml). NO production was measured using Griess reagent. Briefly, the cell supernatant was added into 96 wells for 50 μl/well, followed by inserting 50 μl Griess Reagent I and II. Production of NO was determined by microplate reader at 540 nm.

### 2.7 ELISA analysis

Cell ELISA was performed as its introduction. Briefly, cells in 6‐well plates were pretreated with or without the peptide (25 μg/ml) for 2 hr before stimulation with TNF‐α (10 ng/ml). SOD, GPx, MDA, and iNOS activities in cells were measured using corresponding ELISA assay kit. Cells were digested and fragmented by repeating freeze–thaw three times (5 min in −80°C and 5 min in 37°C) to determine intracellular enzyme activity. Intracellular protein concentration was quantified using BCA protein quantitative kit. The final results of enzyme activity = the actual measured concentration × intracellular protein concentration.

### 2.8 Observation of NF‐κB nuclear translocation

Cells were immunofluorescence stained to detect the main subunit of NF‐κB, p65, to confirm that whether NF‐κB was activated or not. Confluent cell monolayers in 96‐well plates were pretreated with 25 μg/ml peptide for 6 hr prior to stimulation with 10 ng/ml TNF‐α. Cells were fixed for 10 min, blocked for 1 hr, and incubated overnight with primary antibody against p65. The next day, cells were incubated successively with anti‐rabbit Cy3 for 1 hr and DAPI for 5 min in the dark, followed by the addition of sealing fluid to resistance to fluorescence quenching. Under the fluorescence microscope, NF‐κB was dyed in red fluorescence and nucleus in blue. All images presented are in ×200 magnification.

### 2.9 Measurement of nuclear p65 expression

Nuclear fractions were isolated using the Nuclear Protein Extraction Kit (Sainobo Co., Ltd.). Cells (1 × 10^7^ cells) were washed with cold PBS and resuspended in 1 ml of buffer A and incubated on ice. After 20 min, 55 μl of buffer B was added to lyse the cells, which were vortexed for 1 min. Then, cytosolic cell extracts were obtained after centrifuging at 8,500 *g* for 15 min at 4°C. Nuclear were resuspended in 500 μl of buffer C and incubated on ice for 40 min with gentle pipetting every 10 min. Nuclear cell extracts were recovered after centrifugation for 15 min at 8,500 *g* at 4°C. Then, Western blot analysis was performed using the Histone (H3‐antibody (YM3038)) and p65 (NF‐κB p65 (D14E12) rabbit mAb (#8242)) antibodies.

### 2.10 Statistical analysis

All data presented as mean value with standard error (SE) or standard deviation (*SD*). Data are expressed as fold change over the untreated control. One‐way analysis of variance with an appropriate post‐test was used for the determination of statistical significance. *p*‐value <.05 and .01 was defined as statistically significant. All statistical analyses were performed using SPSS software.

## 3 RESULTS

### 3.1 Effects of AAGALPS on viability of TNF‐α‐stimulated HUVECs

**Figure 1:**
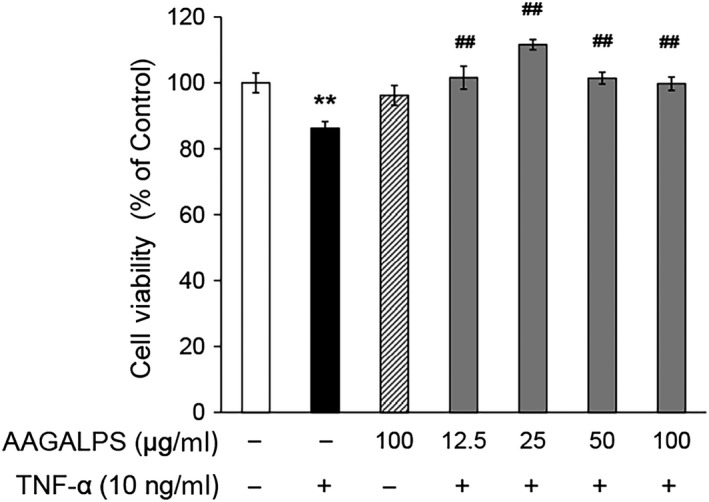
Effects of AAGALPS on viability of TNF‐α‐stimulated HUVECs. Confluent HUVECs were pretreated with different concentrations of AAGALPS for 1 hr prior to the incubation with 10 ng/ml TNF‐α for 6 hr. Cell viability was analyzed by using CCK‐8 according to the standard operation instructions. Cells viability levels are expressed as fold over the control. Bars represent mean values ± SE, *n* = 6. ** indicates *p* < .01 as compared to control; ## indicates *p* < .01 as compared to TNF‐α alone

HUVECs’ viability was measured to evaluate whether the peptide AAGALPS protected endothelial cells from TNF‐α‐induced injuries. As shown in Figure 1, 12 hr of exposure to TNF‐α (10 ng/ml) dramatically decreased the HUVECs’ viability (86 ± 1%) compared with normal control (100%) (*p* < .01). The pretreatment with AAGALPS obviously restored the cell viability and showed the best efficacy at 25 μg/ml (112 ± 2%) (*p* < .01). Although the effect reduced slightly as the growth of concentration to 100 μg/ml, it was still distinct compared with the model group (*p* < .01). At the same time, AAGALPS at 100 μg/ml dose had no conspicuous effect on cell viability in HUVECs (*p* > .05). Meantime, a peptide concentration of only 100 μg/ml was used to detect the toxic effects of the peptide on the cells by treatment at the maximum concentration. The result shows that AAGALPS at 100 μg/ml dose had no conspicuous effect on cell viability in HUVECs (*p* > .05), and it proves that the peptide had no toxic effect on the cells, which is the basis for our next study.

### 3.2 Effects of AAGALPS on TNF‐α‐induced VCAM‐1, ICAM‐1, and MCP‐1 expression

**Figure 2:**
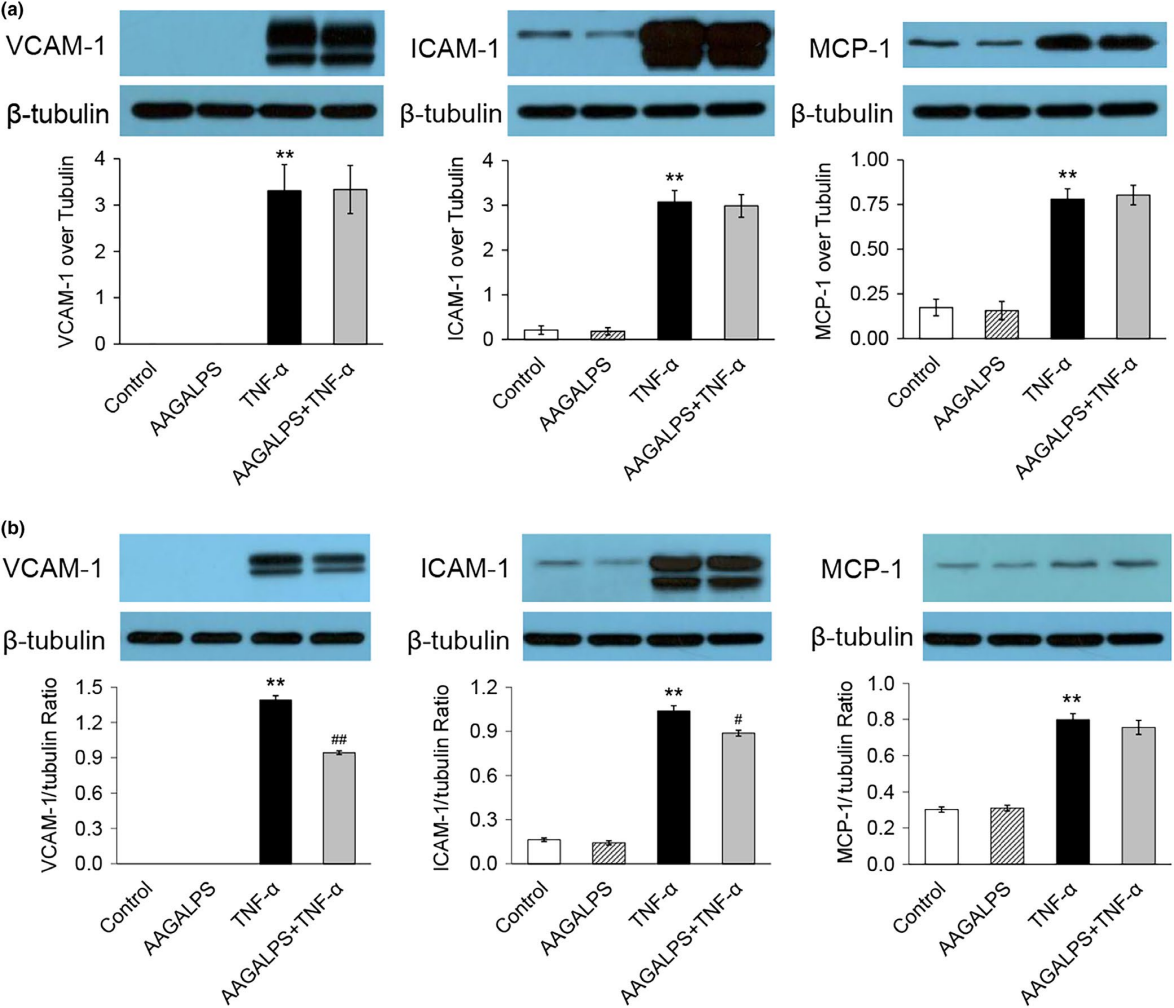
Effects of AAGALPS on TNF‐α‐induced VCAM‐1, ICAM‐1, and MCP‐1 expression. Confluent HUVECs were pretreated with AAGALPS (25 μg/ml) for 2 hr prior to the incubation with 5 ng/ml TNF‐α for 6 hr (a) and pretreated with AAGALPS (25 μg/ml) for 18 hr prior to the incubation with 5 ng/ml TNF‐α for 2 hr (b). VCAM‐1, ICAM‐1, and MCP‐1 protein expressions were measured by Western blot. Bars represent mean values ± SE, *n* = 3. Representative Western blot results are shown. ** indicates *p* < .01 as compared to control; ## indicates *p* < .01 as compared to TNF‐α alone; and # indicates *p* < .05 as compared to TNF‐α alone

To determine whether TNF‐α‐induced VCAM‐1, ICAM‐1, and MCP‐1 production was affected by AAGALPS, HUVECs were pretreated with AAGALPS (25 μg/ml) for 2 hr prior to TNF‐α (5 ng/ml) stimulation for 6 hr. As shown in Figure 2, the cells of control group expressed little ICAM‐1 and MCP‐1, and no VCAM‐1. The TNF‐α stimulation significantly increased the expressions of VCAM‐1, ICAM‐1 and MCP‐1 in HUVECs (*p* < .01). When HUVECs were pretreated with AAGALPS (25 μg/ml) for 2 hr prior to the stimulation with 5 ng/ml TNF‐α for 6 hr, the AAGALPS did not suppress the TNF‐α‐induced overexpression (Figure 2a). Nevertheless, when cells were pretreated with AAGALPS (25 μg/ml) for 18 hr prior to TNF‐α (5 ng/ml) stimulation for 2 hr, AAGALPS markedly suppressed the TNF‐α‐induced expression of VCAM‐1 by more than 30% and expression of ICAM‐1 by almost 15%, while the expression of MCP‐1 was not affected significantly (Figure 2b).

### 3.3 Effects of AAGALPS on TNF‐α‐induced iNOS and NO concentrations

**Figure 3:**
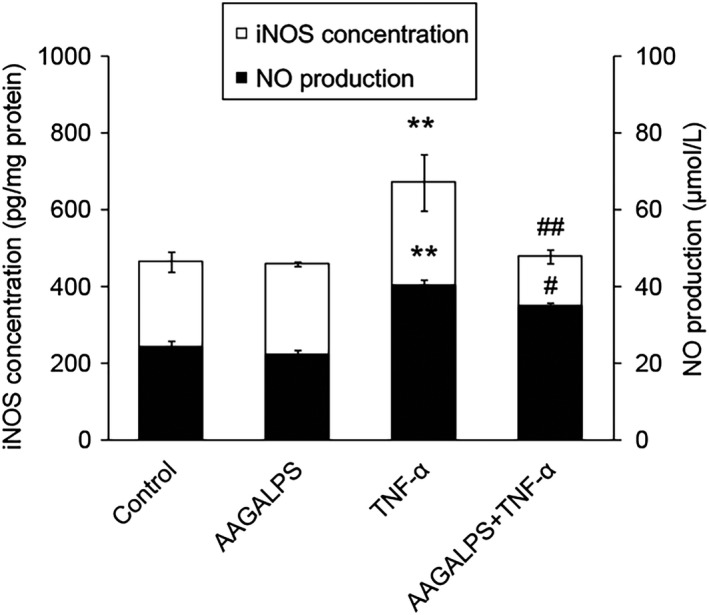
Effects of AAGALPS on TNF‐α‐induced iNOS expression and NO production. Confluent HUVECs were pretreated with AAGALPS (25 μg/ml) for 2 hr prior to the incubation with 10 ng/ml TNF‐α for 12 hr. iNOS activity and NO content in cells culture supernatant were measured using corresponding assay kit. Bars represent mean values ± SE, *n* = 6. ** indicates *p* < .01 as compared to control; ## and # indicate *p* < .01 and *p* < .05, respectively, compared to TNF‐α alone

Compared with the control cells, TNF‐α‐stimulated HUVECs caused about 85% up‐regulation of NO production (*p* < .01) (Figure 3), while the NO production of TNF‐α‐stimulated HUVECs with the treatment of AAGALPS (25 μg/ml) was reduced from 40.1 ± 1.5 μmol/L to 34.7 ± 0.8 μmol/L (*p* < .05). Treatment of AAGALPS (25 μg/ml) alone had no significant effects. To further provide evidence of TNF‐α‐induced NO production in HUVECs, the concentration of intracellular iNOS was measured by ELISA assay kit. As shown in Figure 3, iNOS in cells induced by TNF‐α was about 35% higher than that of control cells, but pretreatment of AAGALPS (25 μg/ml) for 2 hr inhibited the activity from 669.4 ± 73.5 to 476.8 ± 17.8 pg/mg protein (*p* < .01). Simultaneously, there were no obvious differences between cells of control group and AAGALPS group.

### 3.4 Effects of AAGALPS on TNF‐α‐induced oxidative stress

**Figure 4:**
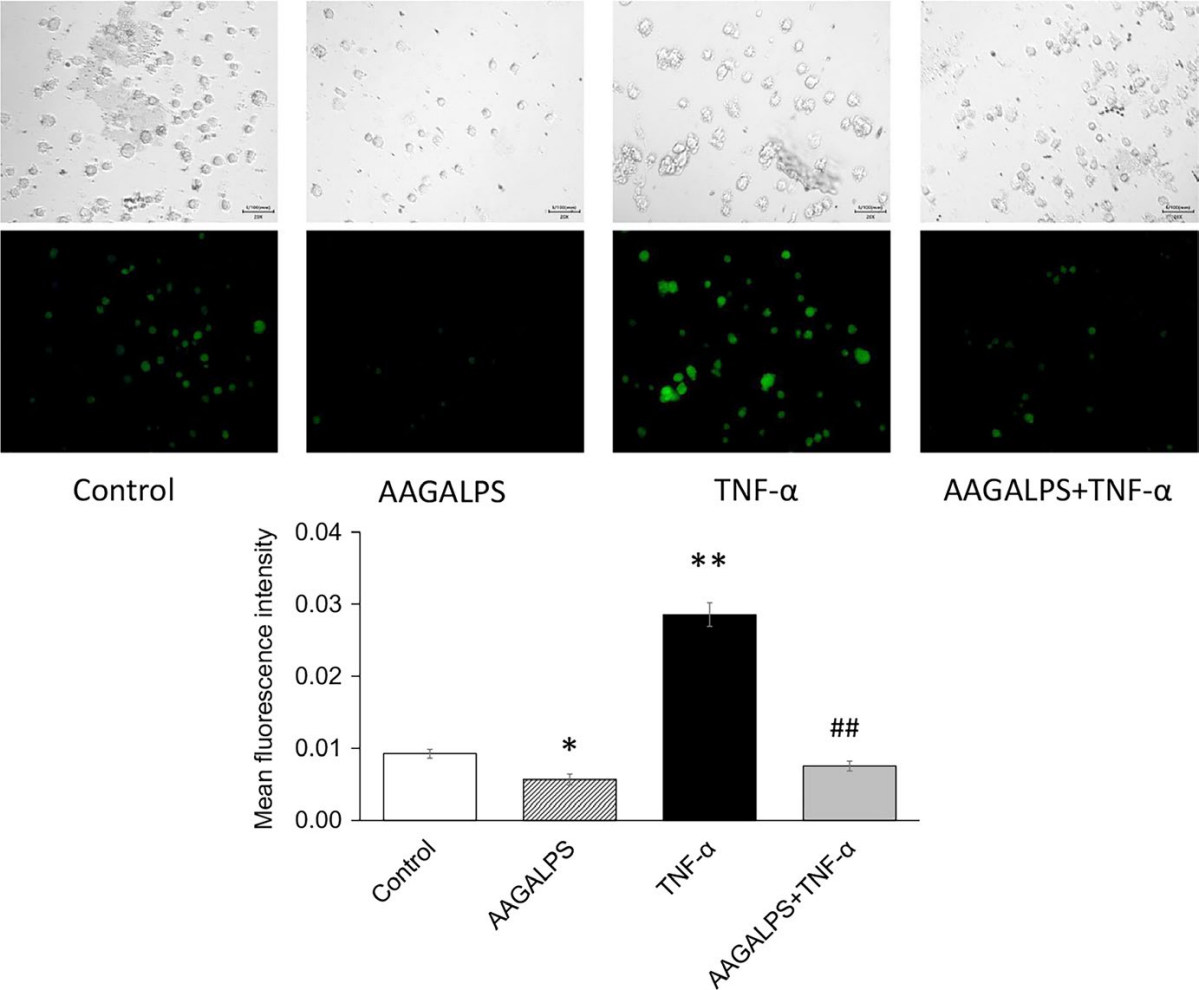
Effects of AAGALPS on TNF‐α‐induced ROS. Confluent HUVECs were pretreated with AAGALPS (25 μg/ml) for 18 hr prior to the incubation with 10 ng/ml TNF‐α for 2 hr, followed by labeled with fluorescent probe DCFH‐DA (10 μM) for 30 min. Images were observed with under a fluorescent microscope at × 200 magnification. The upstairs images under white light were in the same view as the fluorescent images, respectively, to show cell number and location. The mean fluorescence intensity was counted by ImageJ. Bars represent mean fluorescence intensity ± SE, *n* = 5. ** and * indicate *p* < .01 and *p* < .05, respectively, compared to control; ## indicates *p* < .01, compared to TNF‐α alone

To investigate the potential modulation of oxidative stress by AAGALPS, ROS production was evaluated in HUVECs. AAGALPS (25 μg/ml) markedly inhibited the increased ROS production induced by TNF‐α (10 ng/ml) (*p* < .01) (Figure 4). Furthermore, basal ROS production in HUVECs without TNF‐α was also reduced by AAGALPS alone (*p* < .05). Moreover, we also analyzed the effects of AAGALPS on the content of GPx. The AAGALPS resulted in the significant increases in the contents of GPx, and decreases in MDA content with or without TNF‐α stimulation (*p* < .01) (Table 1). However, the peptide did not recover SOD content. It is worth noting that after the peptide treatment, the cells looked like they were getting smaller. But this does not mean that the treatment of the peptide caused cell apoptosis, because we have confirmed that the peptide could not induce the cell apoptosis and did not have cytotoxicity using CCK‐8 kit assay.

**Table 1:** Effects of AAGALPS on GSH‐Px, SOD, and MDA concentritions

	Control	AAGALPS	TNF‐α	AAGALPS + TNF‐α
	*n* = 6			
GSH‐Px (pg/mg protein)	83.7 ± 2.2	132.5 ± 11.0**	65.7 ± 2.4**	100.4 ± 8.9**^##^**
SOD (ng/mg protein)	267.2 ± 5.3	260.1 ± 10.7	229.8 ± 8.0*	236.4 ± 9.8*
MDA (mmol/L)	1.19 ± 0.06	0.61 ± 0.02**	1.71 ± 0.08**	0.75 ± 0.07**^##^**

Note

Means and standard deviation (*SD*) were determined for 6 separate experiments.

** and * indicate *p* < .01 and *p* < .1, respectively, compared to control; ## indicates *p* < .01, compared to TNF‐α alone.

### 3.5 Effects of AAGALPS on inhibited TNF‐α‐activated IKK‐NF‐κB pathway

**Figure 5:**
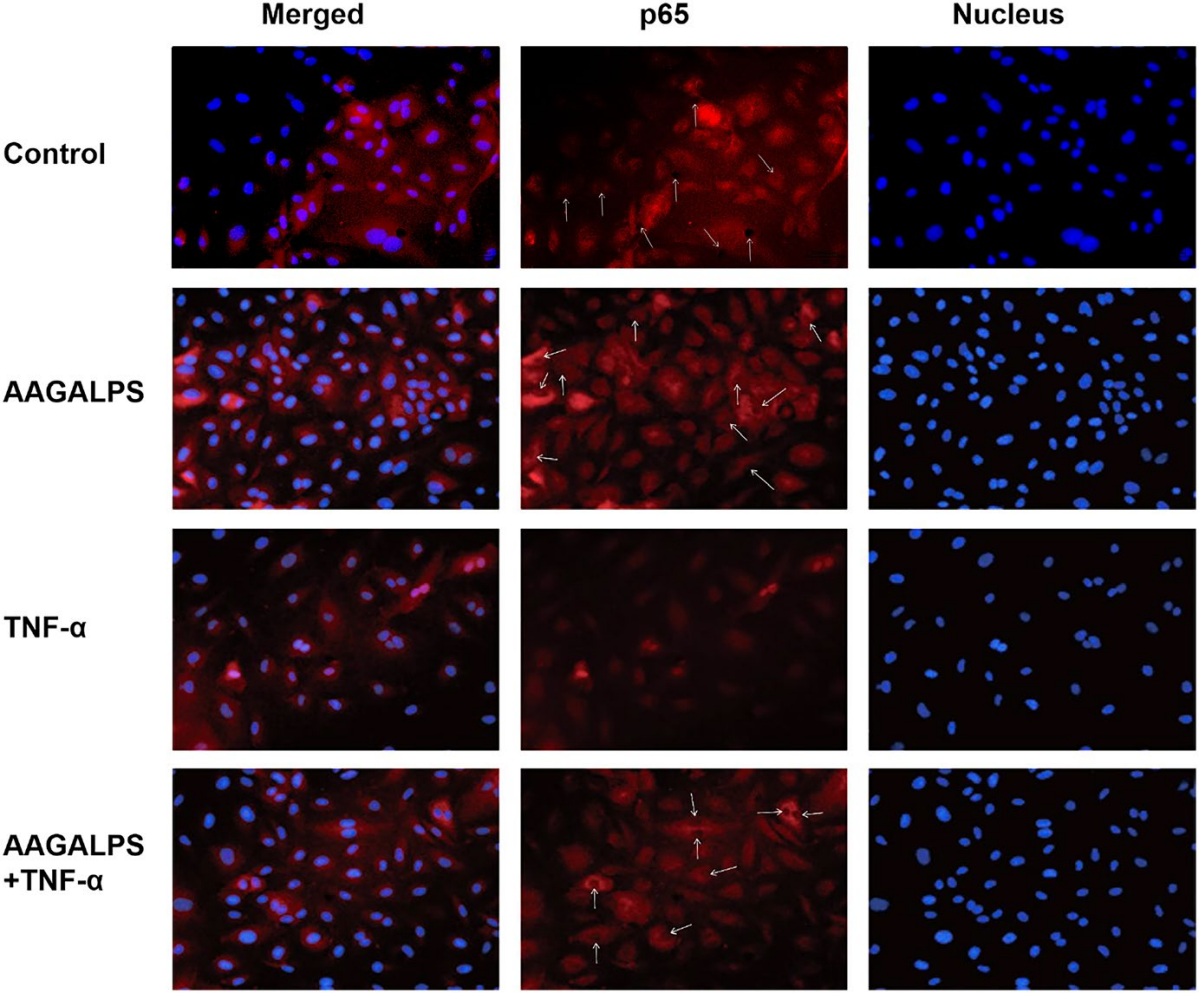
Effects of AAGALPS on TNF‐α‐induced p65 translocation. Confluent HUVECs were pretreated with AAGALPS (25 μg/ml) for 6 hr prior to the incubation with 10 ng/ml TNF‐α for 1 hr. HUVECs were stained with P65 antibody. Nucleus and p65 protein were dyed into blue and red fluorescence, respectively. A representative set of images from three independent experiments was shown

**Figure 6:**
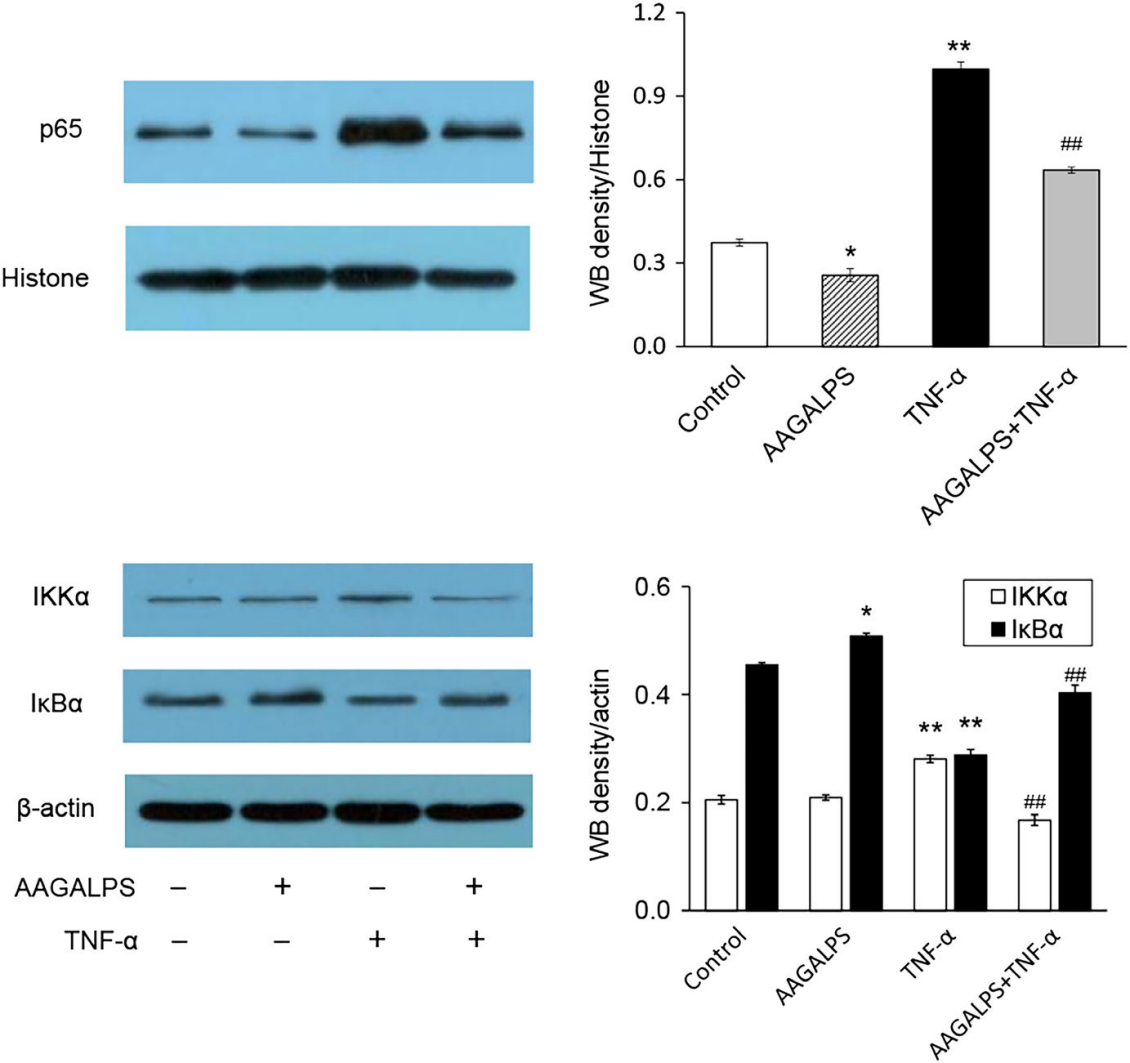
Effects of AAGALPS on inhibited TNF‐α‐activated IKK‐NF‐κB pathway. Confluent HUVECs were pretreated with AAGALPS (25 μg/ml) for 6 hr prior to the incubation with 10 ng/ml TNF‐α for 1 hr. IKKα, IκBα, and nuclear p65 protein expressions were measured by Western blot. Bars represent mean values ± SE, *n* = 3 separate experiments. ** and * indicate *p* < .01 and *p* < .05, respectively, compared to control; ## indicates *p* < .01, compared to TNF‐α alone

HUVECs were treated with AAGALPS for 6 hr and then treated with or without TNF‐α (10 ng/ml) for 1 hr. TNF‐α stimulation caused nuclear translocation of p65. At the same time, pretreatment with AAGALPS abolished the TNF‐α‐induced nuclear localization of p65 (Figure 5). To confirm that AAGALPS played a key role in the IKK‐NF‐κB pathway, levels of IKKα, IκBα, and nuclear p65 protein were also evaluated. As shown in Figure 6, the amount of IKKα was greatly increased by the TNF‐α stimulation, while AAGALPS significantly suppressed the increase, and it also inhibited the degradation of IκBα accordingly (*p* < .01), as well as nuclear p65 protein expression (*p* < .01). The decreased protein expression of nuclear p65 confirmed the results of Figure 5 that AAGALPS suppressed translocation of p65 from cytosol to nuclear.

## 4 DISCUSSION

The excessive inflammation is an essential factor for the origination of atherosclerotic lesion when endothelial cells are stimulated by pro‐inflammatory cytokines such as TNF‐α, ox‐LDL, and IL‐6, to express the NO, VCAM‐1, ICAM‐1, MCP‐1 etc. Therefore, TNF‐α‐induced HUVEC injury model was used to analyze the protective effects of AAGALPS on vascular endothelial cells. The result showed that viability of HUVECs damaged by TNF‐α rose notably with AAGALPS (12.5, 25, 50, and 100 μg/ml), which revealed that the peptide contributed to protecting vascular cell from injury (Figure 1). This finding indicated that the peptide AAGALPS from rice α‐globulin was directly responsible for its antiatherogenic effect.

ICAM‐1 and VCAM‐1 are both significant in excessive inflammation and play vital roles in inflammatory cell adhesion and cell signal conduction (Chai et al., 2008). Both ICAM‐1 and VCAM‐1 accelerate the development of various diseases such as atherosclerosis. Particularly, VCAM‐1 is one of the primary endothelial adhesion molecules that play a vital role in the aggregation of monocytes to the vascular endothelium, which is a crucial process during the development of atherosclerosis (Cybulsky & Gimbrone, 1991). MCP‐1 is a pivotal chemokine in vascular illness, and the expression of MCP‐1 has been detected in many tissues during inflammation‐reliant diseases, including atherosclerosis (O'Hayre, Salanga, Handel, & Allen, 2008). In addition, NF‐κB is critical to TNF‐α‐stimulated VCAM‐1 and ICAM‐1 expression (Zhu et al., 2013). In the present study, 2‐hr pretreatment of AAGALPS was too short to affect the expression of the adhesion molecules at 25 μg/ml significantly, while when we lengthened the pretreatment time and reduced the time of TNF‐α stimulation, the peptide suppressed the up‐regulation of VCAM‐1 and ICAM‐1 in HUVECs stimulated with TNF‐α, while it had no significant effect on overexpression of MCP‐1 proteins (Figure 2b). In addition, when cells were pretreated with AAGALPS (25 μg/ml) for 18h prior to TNF‐α (5 ng/ml) stimulation for 2h, AAGALPS markedly suppressed the TNF‐α‐induced expression of ICAM‐1 by almost 15%. When HUVECs were pretreated with AAGALPS (25 μg/ml) for 2 hr prior to the stimulation with 5 ng/ml TNF‐α for 6 hr, the AAGALPS did not suppress the TNF‐α‐induced overexpression, and this may because the peptide incubation time is too short and the TNF‐α stimulation time is too long, causing the peptide to fail to function. The optimal time for peptide incubation and TNF‐α stimulation may be used as a research point in the subsequent process.

Nitric oxide (NO) as an indicator of inflammation is produced by nitric oxide synthases (NOS) which performs in three hypotypes, namely iNOS, eNOS, and nNOS. Only iNOS raises NO expression induced by pro‐inflammatory cytokines, such as TNF‐α (Ruimi et al., 2010). iNOS is induced by the atherosclerotic plaque of vascular cells, and its activity results in high local concentrations of NO (Wang et al., 1999). It is generally known that oxidative stress can induce endothelial cell apoptosis through NF‐κB‐iNOS‐NO signaling pathway, and ROS is an important parameter of oxidative stress. So we measured the NO and iNOS indicators to identify potential signaling pathways that peptide protect endothelial cells from oxidative stress. As a result, AAGALPS significantly reduced the values of NO and iNOS in TNF‐α‐injured HUVECs (Figure 3). These consequences revealed that the peptide improved the injury of TNF‐α‐induced vascular endothelial cells by inhibiting excessive inflammation.

As noted before, increased oxidative stress plays a key role in the pathogenesis of cardiovascular diseases (Landmesser & Harrison, 2001). The stability of ROS such as superoxide anion, hydrogen peroxide, and hydroxyl radicals is important in redox balance (Aw, 1999). Scientists have generally regarded these elementary molecules as harmful to the vasculature, leading to pathological processes, such as hypertension, restenosis, and atherosclerosis. In the present study, AAGALPS pretreatment for 2 hr could inhibit TNF‐α‐induced ROS production in HUVECs. Moreover, it was shown that the content of GPx was significantly increased by AAGALPS, which were kinds of enzyme involved in the detoxification of ROS. GPx can catalyze the redox between peroxide and GSH which transforms peroxide into non‐toxic alcohols (Yamada et al., 2010). Previous experiments reveal that free radical‐mediated lipid peroxidation can induce endothelial cell injury and dysfunction (Hennig & Chow, 1988). MDA is an important index of evaluating the degree of lipid peroxidation. In the present study, AAGALPS suppressed production of MDA significantly, indicating that the peptide inhibited lipid peroxidation induced by TNF‐α. Therefore, AAGALPS has the potential benefit of preventing TNF‐α‐induced vascular endothelial cell injury by ameliorating oxidative stress.

When it comes to the pathway by which TNF‐α stimulated the iNOS, Umansky et al. (Zhu et al., 2013) provided a mechanism for a self‐amplifying signal in the inflammatory response, since the iNOS in endothelial cells is regulated by NF‐κB. NF‐κB activation could be regulated by cytoplasmic proteins IκBα. IκBα inhibits NF‐κB activity by forming an inactive complex. Once IκBα is activated by IKKα, it will degrade rapidly and the p65 protein will be exposed and transfer to nucleus, a promoter, which activates certain genes related with inflammatory (Aw, 1999). Simultaneously, NF‐κB is a main target for ROS, and its activation has been linked to endothelial cell dysfunction and survival (Deshpande, Angkeow, Huang, Ozaki, & Irani, 2000).

Therefore, we tried to confirm that whether the peptide had beneficial effects on NF‐κB pathway. As a result, AAGALPS was able to inhibit the IKK‐NF‐κB pathway and nuclear translocation of p65 (Figure 5). In addition, AAGALPS conduced to index of p65, IKKα, and IκBα protein in HUVECs injured by TNF‐α (Figure 6). These results suggested that the AAGALPS of rice globulin peptide could prevent TNF‐α‐induced HUVEC injury by inhibiting cellular oxidative stress; therefore, cell inflammation could be reduced. The reduction of oxidative stress in this cell is due to the inhibition of the IKK‐NF‐kB signaling pathway.

## 5 CONCLUSIONS

In conclusion, these results in the present study revealed that AAGALPS (25 μg/ml) attenuated TNF‐α‐induced injury by restraining inflammatory responses and oxidative stress in HUVECs, relating to suppression of NF‐κB pathway. Therefore, these results revealed that AAGALPS as one of the bioactive peptides lay behind the antiatherosclerosis effects of rice α‐globulin.

## Conflict of Interest

The authors declare that they do not have any conflict of interest.

## Ethical Approval

This study does not involve any human or animal testing.

## Informed Consent

Written informed consent was obtained from all study participants.
